# Sensory Processing Sensitivity Questionnaire: A Psychometric Evaluation and Associations with Experiencing the COVID-19 Pandemic

**DOI:** 10.3390/ijerph182412962

**Published:** 2021-12-08

**Authors:** Klara Malinakova, Lukas Novak, Radek Trnka, Peter Tavel

**Affiliations:** 1 Olomouc University Social Health Institute, Palacký University Olomouc, 771 11 Olomouc, Czech Republic; lukas.novak@oushi.upol.cz (L.N.); trnkar@volny.cz (R.T.); peter.tavel@oushi.upol.cz (P.T.); 2 Department of Science, Prague College of Psychosocial Studies, Hekrova 805, 149 00 Prague, Czech Republic

**Keywords:** high sensitivity, sensory processing sensitivity, measurement, psychometric evaluation, COVID-19

## Abstract

Sensory processing sensitivity (SPS) is a common human neurobiological trait that is related to many areas of human life. This trait has recently received increased public interest. However, solid scientific research on SPS is lagging behind. Progress in this area is also hindered by a lack of comprehensive research tools suitable for a rapid assessment of SPS. Thus, the aim of this study was to offer a newly developed tool, the Sensory Processing Sensitivity Questionnaire (SPSQ), and to assess its psychometric properties and associations with emotional and relational variables measured during the first wave of the COVID-19 pandemic. We found the tool to have good psychometric characteristics: high temporal stability (*r* = 0.95) and excellent internal consistency (Cronbach’s α = 0.92; McDonald’s ω = 0.92). The fit of the SPSQ bi-factor model was satisfactory: *χ*^2^ (88.0) = 506.141; *p* < 0.001; CFI = 0.993; TLI = 0.990; RMSEA = 0.070; SRMR = 0.039. Testing of configural, metric, scalar and strict invariance suggested that the SPSQ assesses SPS equivalently between males and females. The scale’s validity was supported via a strong association with an existing SPS measure. Further, we observed higher total SPSQ scores among women, students and religious respondents, and we found that more sensitive respondents reported higher feelings of anxiety and more deterioration in relationships during the COVID-19 pandemic. Thus, this study also identifies people with this trait as being potentially more vulnerable during periods of an increased presence of global stressors.

## 1. Introduction

Sensory processing sensitivity (SPS) is defined as a personal disposition to being sensitive to subtle stimuli and being easily over-aroused by external stimuli [1]. It has recently been proposed as a human neurobiological trait found to be significantly higher in 10–20% of the population [2]. This increased sensitivity of the central nervous system means individuals with this trait are able to process information deeper than usual [3] and are more prone to arousal, especially after exposure to sensory stressors, such as bright lights, loud noise, strong smells and dense and chaotic environments [2]. Besides physical stressors, it is also related to a deeper processing of mental, social and emotional stimuli [3]. 

While this high reactivity has its positive aspects, it also has downsides. Studies in the area of mental health show that Highly Sensitive Persons (HSPs) represent a minority in society. However, their occurrence among patients in most practices is likely close to 50% [4]. SPS has been associated with higher levels of stress [5], anxiety and depression [6], low self-esteem [7], agoraphobia [8], negative affectivity and heightened shyness [9] and attention-deficit hyperactivity disorder (ADHD) [10]. HSP employees were found to be more sensitive to work stress, work displeasure and the need for recovery [11]. HSPs are also influenced more than other people by adverse childhood experiences and poor parenting [6].

However, research has also revealed that SPS is more a variation in information processing than a disorder [12], and the abovementioned personality traits and mental health issues show only a partial overlap with SPS [13]. In fact, though HSPs are at a greater risk for maladjustment in negative environments [14], they also react strongly to positive stimuli [15]. Thus, SPS also has its bright side, and when expressed adaptively, it correlates with better perception, inventiveness and imagination [16]; higher sensitivity to art and creativity [17]; openness to experience [13]; better decision-making; and engagement in meaningful work [18]. Many HSPs are empathic, nurturing and intuitive [12].

In their critical review, Greven et al. [19] pointed to the important fact that although SPS is receiving increased interest among the general public and in the media, basic scientific research in this area is not keeping pace. The authors stated that this imbalance between the demand for information from society and the scientific knowledge gathered thus far can lead to misinterpretations of SPS characteristics. However, research in this area is also complicated by a lack of reliable, valid and comprehensive assessment tools. 

Currently, the Highly Sensitive Person Scale (HSPS) developed by Aron [1], also the founder of the whole concept, is predominately used for assessing sensory processing sensitivity. This scale has passed several scientific validations [20]. The authors of the scale indicate a one-factor solution; however, Lionetti et al. [21] suggested a bi-factorial solution, where the HSPS was made up of both a general sensitivity construct and three individual subscales. Nevertheless, other authors came to even different conclusions, so at the moment, there is no agreement regarding the internal structure of sensitivity [22]. Moreover, this instrument is relatively lengthy: it includes 27 self-reporting questions, some of which are rather repetitive. At the moment, the scale is being reviewed by the authors [12], and also a shortened 12-item version is in preparation. As a part of a further development, the HSPS was also adapted as a 12-item version for children [23]; however, there are questions regarding the comparability of both instruments [13].

Besides the HSPS, sensory aspects of SPS in adults are also assessed by the Adolescent/Adult Sensory Processing [24], a 60-item measure assessing behavioral responses to everyday sensory experiences. However, this measure did not show a consistent factor structure when adapted to other languages [25]. The Sensory Perception Quotient [26], the Sensory Hypersensitivity Scale [27] and the Sensory Sensitivity Scales [28] belong amongst other instruments measuring sensory aspects. Other aspects can also be measured by the Questionnaire Measure of Stimulus Screening and Arousability [29] and by the Sensory Discomfort and Orienting Sensitivity subscales of the Adult Temperament Questionnaire [30]. Nevertheless, these instruments do not capture all SPS traits (e.g., its emotional aspects). Most importantly, many of the available instruments are lengthy and all of them are based on statements describing situations and experiences of HSPs. Therefore, using these tools could be time consuming and consequently inconvenient for their use in larger surveys or situations where a rapid assessment is needed, i.e., in the work of a physician or a psychiatrist. Thus, there is a need for a valid and reliable tool that would also be comprehensive enough for use in these conditions. We tried to address this gap by offering an instrument that covers the main aspects of SPS while using a minimal amount of words.

In our study, we decided to also include measures of neuroticism and religiosity. Aron [4] points to the fact that, though neuroticism correlates with SPS, we have to distinguish between these separate constructs. Therefore, the comparisons among different sociodemographic groups were also controlled for neuroticism. Religious affiliation was assessed as a part of the background characteristics of the sample, because associations of SPS with religiosity/spirituality are often observed by psychotherapists [4]. However, these associations are not sufficiently explored in scientific articles. The last part of the assessment focused on the association of SPS with variables measured during the first wave of the COVID-19 pandemic. This pandemic represents a period of a heightened uncertainty and fear in the general population. Past research has demonstrated that anxiety and post-traumatic stress disorder symptoms occur to a higher degree in the general public during a pandemic crisis [31,32]. Given the existential threats that a pandemic elicits, global anxiety and heightened stress represent serious consequences of this traumatic event. Fear of becoming infected, fear for the health and lives of significant others, fear of the socioeconomic consequences of the pandemic, fear of the unavailability of healthcare, and fear of an insufficient food supply are examples of alarmed and anxiety-related responses to the COVID-19 crisis [33,34]. Therefore, because of their possible high chronic self-perceived stress and high trait anxiety, highly sensitive individuals may be extremely vulnerable to maladaptive mental health outcomes during a serious global crisis, such as the COVID-19 pandemic. 

Thus, the development of a brief and easy-to-administer tool for psychometric assessment of SPS is both timely and vital. This pre-registered study (see https://osf.io/kd9bh; accessed on 7 December 2021) describes a new tool, the Sensory Processing Sensitivity Questionnaire (SPSQ), and describes its psychometric properties as well as selected associations with experiencing the COVID-19 pandemic. 

## 2. Materials and Methods

### 2.1. Participants and Procedure

A sample of the Czech population aged eighteen years and older was obtained through two online surveys which were part of broader research and included data on high sensitivity. In addition, the first study also focused on the experiences of people during the first outbreak of the pandemic. Data were collected by a professional agency (the The Czech National Panel, Prague, Czech Republic) during the first wave of the COVID-19 pandemic (April and May 2020). The second study followed shortly afterwards (May and June). The online surveys were prepared at the researcher’s institution, and the agency ensured their distribution by using quota sampling to achieve a balanced sample in terms of age and gender. 

Data from both samples were merged into one dataset (first sample). For the purposes of test–retest reliability, we also recruited a small sample of participants online, using the snowball technique (second sample). Data from both samples were consequently checked for quality. To ensure the high quality of the data, the following exclusion criteria were applied: (1) extremely short time filling out the survey (i.e., less than 15 min for a survey that lasted around 45 min), which would not allow respondents to fill out the survey thoughtfully; (2) inconsistency in repeated control questions regarding participants’ weight (i.e., 2 kg and over), height (2 cm and over) or age (2 years and over); and (3) a unified pattern of responses, i.e., responding to a large number of items in the same fashion. Thus, after excluding problematic respondents (*n* = 27), the first sample consisted of 1919 respondents (mean age 49.4, SD = 16.6; 49.6% men) and the second sample 30 respondents (mean age 29.23, SD = 11.25, 23.33% men).

At the beginning of the survey, participants received written information on the aim of the study and the anonymized handling of data and were made familiar with the online platform for filling out the survey. Before starting the survey, respondents had to explicitly express their informed consent to their participation in the study. Participation in the survey was fully voluntary, so the respondents could stop responding at any time before or during the survey. The study design was approved by the Ethics Committee of the Olomouc University Social Health Institute, Palacký University Olomouc (No. 2021/3).

### 2.2. Measures

High sensitivity was assessed by using the newly developed Sensory Processing Sensitivity Questionnaire (SPSQ), as shown in the Supplementary Materials. In the version originally used in the survey, this scale consisted of 20 items designed on the basis of empirical studies and after consultation with experts in psychological, psychosomatic and psychotherapeutic practice to cover different aspects of this trait. The items were developed along two areas of high sensitivity described in the scientific literature, which resulted in two subscales of the SPSQ Scale: a Sensory Sensitivity Subscale (sensory processing sensitivity) and Other Sensitivity Subscale, which assesses sensitivity to emotions and various life experiences. 

The instruction and wording of the items rests on the assumption that highly sensitive people are usually aware that they are more sensitive to certain stimuli than other people, as this is also the feedback they have been receiving from the world during their lifetime. To achieve maximum compatibility and shortness of the scale, the items are not formulated as statements but mostly as single words covering the key areas of high sensitivity. Thus, the introductory question was formulated as follows: “Please indicate to what extent you think that compared to other people you are sensitive to the following stimuli”. The main anchor points of the answering scale were labeled as follows: “0 = compared to others, I am not sensitive to them at all”; “5 = about the same as the people around me”; and “10 = much more sensitive than the people around me”. This introductory question was followed by a set of 10 sensory component items (light; sounds; smells; taste; tactile stimuli, e.g., touch, clothing, etc.; pain; hunger; caffeine and other stimulants; heat; and cold) and a set of 10 other sensitivity items (your emotions; emotions of other people; sudden changes; your inner world; the need to do many things at once; lack of privacy; stay in nature; criticism; the need for harmony in life; and the need to make decisions).

As a measure of concurrent validity, we used the Highly Sensitive Person Scale (HSPS) proposed by Aron and Aron [1]. This scale is a self-report instrument consisting of 27 items that focus on different areas of high sensitivity. Response possibilities for these items are on a 7-point scale that ranges from “Not at all” (1) to “Extremely” (7), leading to scores from 27 to 189. A higher score represents higher sensitivity. Cronbach’s alpha was 0.91 in our sample.

*Neuroticism* was assessed by using the Neuroticism Subscale of the Big Five inventory [35]. This subscale consists of eight statements (three of them are reverse-worded). Participants report the degree of their agreement with each statement on a 5-point Likert scale ranging from “disagree strongly” (1) to “agree strongly” (5), producing scores from 8 to 40. A higher score represents higher neuroticism. Cronbach’s alpha was 0.87 in our sample.

*Experience of a COVID-19 pandemic* was assessed by using an introductory question: “Has anything changed in your life in connection with the pandemic in the following areas?”, followed by a set of nine items focusing on relationships (with a partner, children and other people in the household), feelings (loneliness, threat, fear and anxiety, helplessness, hope) and the day structure. Possible answers to these items were: “has worsened (−1)”, “has not changed (0)” and “has improved (1)”. The answers were further dichotomized: 0 and 1 were classified as “has not worsened” and -1 as “worsened”.

*Religious affiliation* was measured by the question: “At present, would you call yourself a believer?” with possible answers: yes, I am a member of a church or religious society; yes, but I am not a member of a church or religious society; no; and no, I am a convinced atheist. 

The other background characteristics, i.e., gender, age and other basic sociodemographic characteristics, were obtained by means of the questionnaire. 

### 2.3. Statistical Analyses

The normality of the distribution was assessed by using histograms and Mardia’s test of skewness and kurtosis. As the normality assumption was violated, non-parametric methods were used. Equality of variances was examined by visual inspection of the residual plot, suggesting homoscedasticity of the SPSQ data. The presence of outliers was detected by Median Absolute Deviation (MAD). No missing data were present. 

To explore the stability of the factor solution, the first sample (*n =* 1919) was randomly divided into two halves. On the first half (a calibration sample, *n* = 959), the Exploratory Factor Analysis (EFA) was performed, whereas the second (a validation sample, *n* = 960) was assessed by using Confirmatory Factor Analysis (CFA). In the first stage of the development of the SPSQ, when the item pool was created, EFA was conducted to find out what items should remain in the scale. The EFA was calculated based on the polychoric correlation matrix with oblimin rotation. The Weighted Least Squares (WLS) estimator was used. In the EFA, items were excluded from the item pool under the following conditions: (1) absence of loading to any factor; (2) loading was not theoretically meaningful; (3) communalities (h2) ≤0.4; (4) cross-loading was ≥0.30 and (5) factor loading ≤0.4. Items were considered as forming a factor if: (1) the factor has a theoretical justification; (2) the factor was formed by ≥3 items; (3) alpha reliability of the factor was ≥0.7 [36]. The number of factors to extract was determined by three methods: (1) parallel analysis (PA) [37], (2) the Hull method [38] and (3) the comparison data (CD) method [39]. The PA was based on 5000 matrices of randomly generated data. The EFA was performed in the psych package [40] in R [41]. 

In the next step, a CFA run on a polychoric correlation matrix was performed. In the CFA, the model fit was explored by using (1) the Tucker–Lewis index (TLI) and (2) the comparative fit index (CFI), (3) the Root Mean Square Error of Approximation (RMSEA) and (4) the Standardized Root Mean Square Residual (SRMR). Finally, the Chi-Square (*χ*^2^) statistic was also calculated to evaluate model fit. Values of TLI and CFI > 0.95 indicate an acceptable fit [42] and >0.97 a good fit [43]. Values of SRMR and RMSEA < 0.08 reflect an acceptable fit [44] and <0.05 a good fit [45]. The CFA models were fitted by the Diagonally Weighted Least Squares estimator (DWLS). The chi-square difference test with Satorra Bentler correction was used to explore nested models. The CFA was calculated by using the lavaan [46] package in R.

The internal consistency of the scale was evaluated by Cronbach’s alpha, McDonald’s omega and item-total correlations. All internal consistency measures were calculated in R packages psych [40] and ufs [47]. 

The Mann–Whitney U test was used for comparing the two groups (religious affiliation and gender). The Kruskal–Wallis rank sum test was conducted for comparing multiple groups (education, economical status and family status). The Games–Howell test with Bonferroni correction was calculated for post hoc testing (Education). Binary logistic regression was calculated to assess the relationship between deteriorated feelings, deteriorated relationships and deteriorated structure of the day during the COVID-19 pandemic. The regression analysis was conducted in the following steps: First, a model with SPSQ total score as a predictor was fitted. In the second step, the SPSQ total score was replaced by the SPSQ Sensory Sensitivity (SS) Subscale score and the model was fitted again. The same process was performed in models containing covariates. After both crude and adjusted effect were estimated, the dependent variable was replaced and the described steps described were repeated. The independent variable was standardized to Z-score. Covariates in the adjusted effect consisted of age, gender and socioeconomic status. Measurement equivalence (configural, metric, scalar and strict) between males and females was examined. Invariance of the measurement was supported if Δ in CFI was <0.01 between invariance models.

## 3. Results

### 3.1. Exploratory Factor Analysis (ERA) Results

The results of Bartlett’s test of sphericity $\chi^{2}$(190) = 22,421.93, *p* < 0.001 and KMO (0.95) indicated that the data are factorable and adequately correlated. The item-to-item correlation of SPSQ items calculated on the first sample ranges from low to high (0.2–0.71). 

The EFA was conducted on the calibration sample (*n =* 959). In the first analysis, we extracted four factors by the PA and two factors by the Hull and CD method. In the EFA, we tested a number of factors extracted by the previously mentioned methods, along with a one-factor solution resulting from theoretical assumptions on the SPS. The first EFA suggested that the item “caffeine and other stimulants” yielded low communalities (0.26–0.30) across the solutions or formed its own factor. For this reason, this item was excluded. In the second analysis, four, two and five factors were extracted, respectively. The item “stay in nature” from the Other Sensitivity Subscale (O) yielded across the solutions a suboptimal $h^{2}$ (around 0.30) and therefore was also excluded from further analysis. In the third EFA, three, two and seven factors were extracted. The item “lack of privacy” had an insufficient $h^{2}$ (around 0.35) in most of the solutions or formed its own factor. Therefore, we excluded this item from the next analysis. During the fourth extraction, three, two and six factors were suggested. In the EFA 4, we found that the item “pain” yielded relatively suboptimal communalities and was thus excluded from the analysis. In the last EFA, all methods except the CD suggested a two-factor structure. Although the items “hunger” and “cold” did not reach $h^{2}$ > 0.40, it was decided to keep these items in the scale, as the scale content validity would be weakened after the exclusion of these items. The two-dimensional version with correlated factors was theoretically meaningful, with overall adequate factor loadings and communalities (Table 1). Correlation between the factors was *r* = 0.63.

### 3.2. Confirmatory Factor Analysis (CFA) Results

CFA was conducted on the validation sample (*n* = 960). Three solutions were tested: (1) a one-factor model; (2) two correlated factors, i.e., Sensory Sensitivity (SS) and Other Sensitivity (OS), suggested by the EFA; and (3) a bi-factor model. In the first model, absolute fit indices suggested that the one-factor model did not reach acceptable fit criteria (see Table 2), with factor loadings ranging from 0.58 to 0.75. Compared to the first model, the two-factor model yielded higher goodness of fit indices (Table 2) and factor loadings (0.68–0.79).

**Figure 1:**
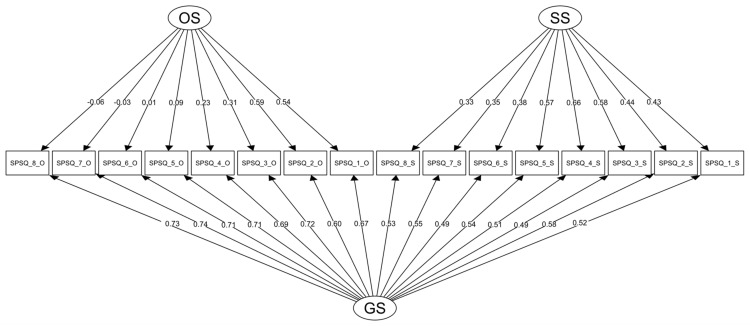
SEM plot depicting factor loadings of the SPSQ items in the bi-factor model. Note: OS = Other Sensitivity, SS = Sensory Sensitivity, GS = General Sensitivity, O = item from Other Sensitivity Subscale, S = item from Sensory Sensitivity Subscale.

The correlation between the two factors was strong, with *r* = 0.70. To further explore the relationship between the two factors, we tested the influence of both subscales on the manifest variables when the effect of the General Sensitivity (GS) factor was taken into account. This bi-factor model yielded from satisfactory to excellent fit indices (see Table 2) and from medium to high factor loadings in the GS factor (0.49, 0.74), see Figure 1. Manifest variables $R^{2}$ yielded acceptable values (0.39–0.74). The chi-square difference test with Satorra-Bentler correction further supported the superiority of the bi-factor model over the one-factor model: $\chi^{2}$(16) = 1041.08; *p* < 0.001 and the two-factor model $\chi^{2}$(15) = 303.12; *p* < 0.001. In the bi-factor model, medium to high factor loadings of the SS items (0.33–0.66) seem to support the domain-specific character of the sensory sensitivity construct. Thus, it is possible to score the SS Subscale separately. To increase the statistical power, further analytical procedures were conducted on the undivided sample (*n* = 1919). 

### 3.3. Reliability

The internal consistency of the entire 16 item scale was excellent: α = 0.92, 95% CI [0.92, 0.93]; $\omega_{t}$ = 0.92, 95% CI [0.92, 0.93]; $\omega_{h}$ = 0.72. Only slightly decreased values were observed in the SS Subscale: α = 0.89, 95% CI [0.88, 0.89]; $\omega_{t}$ = 0.89, 95% CI [0.88, 0.89]. Item–total correlation was adequate, suggesting that the homogeneity of the SPSQ items is sufficient (0.58–0.73; see Table 3). The replicability index of the SS Subscale reached 0.73 (0.92 in the GS factor, respectively). For descriptive statistics of the SPSQ items, see Table 3. 

### 3.4. Measurement Invariance

Results of the measurement invariance testing are presented in Table 4. It was revealed that Δ in CFI between configural, metric, scalar and strict models was not significantly different. Thus, measurement equivalence of the SPSQ between males and females is supported.

### 3.5. Sociodemographic Results

Characteristics of the first sample and differences between sociodemographic groups in the SPSQ total score and SS Subscale are reported in Table 5. In the retest sample (*n* = 30), most of the participants were employed (33.3%), had finished high school (56.7%), were non-religious (60%) and were not in a relationship (76.7%).

The Wilcoxon rank-sum test indicated a higher SPSQ total score and an SS Subscale score in females compared to males. Furthermore, respondents who attended religious services once a week or more have a significantly higher SPSQ total score and an SS Subscale score, compared to those who attended less than once a week. The Dunn test revealed significant difference in the SPSQ total score in students compared to employed respondents and in the SS Subscale in students compared to both employed and pensioners. The Games–Howell test indicated that college students have a higher SPSQ total score compared to those who reached high school without graduation or vocational school. Finally, we found a higher SPSQ total score and an SS score in religious compared to non-religious persons. No other significant effects of sociodemographic variables on the SPSQ score were found.

### 3.6. Differences between Males and Females in SPSQ Scores, Concurrent Validity and Test–Retest Reliability

To verify our assumption regarding the absence of a group difference between males and females in the SPSQ scores, an analysis of the covariance (ANCOVA) was performed. The results revealed that females reached significantly higher SPSQ total scores, even when neuroticism, age and education were statistically controlled: b=0.49, 95% CI [0.35, 0.62], $t\left(1887\right)=7.00$, p<0.001. However, the effect size of this difference was small, with $\eta_{p}^{2}$ = 0.05, 95% CI [0 03, 0.06]. These findings were supported by a robust version of ANCOVA. The results of the correlation analysis indicated a strong positive relationship between the HSPS and the SPSQ: $r_{s}$ = 0.61, *p* < 0.001. The intraclass correlation coefficient revealed that the SPSQ score is stable over time (after a one-week interval): *r* = 0.95, 95% CI [0.9–0.98], *p* < 0.001.

### 3.7. Associations of the SPSQ with Emotional and Relational Variables Measured during the COVID-19 Pandemic

The results of regression analysis exploring associations between the SPSQ scores and emotional, relational and day structure changes linked to the COVID-19 pandemic are presented in Table 6. Both the total and sensory subscale scores were used. The results indicate that after controlling for age, education and socioeconomic status, with one standard deviation increase in the SPSQ total score, the odds of stronger anxiety and fear increased by 68% (*p* < 0.001). A similar but weaker relationship was found between experiencing fear and anxiety and the SS Subscale: 44% (*p* < 0.001). In crude effect, with one standard deviation increase in the SPSQ total score, the odds of deterioration of a relationship with a partner increased by 68% (*p* < 0.001).

## 4. Discussion

The aim of this paper was to offer a new tool for measuring sensory processing sensitivity, the Sensory Processing Sensitivity Questionnaire, to psychometrically assess its characteristics on a large sample of Czech adults and to assess associations of high sensitivity measured by the SPSQ with experiencing the first wave of the COVID-19 pandemic. The EFA suggested the elimination of four items from the original scale, resulting in the 16-item final version of the SPSQ. The results of the CFA and reliability analyses suggested that the scale could either be used as a whole or that the Sensory Sensitivity Subscale could be used separately. Regarding sociodemographic differences, we found that women reported significantly higher levels of SPS than men. Moreover, we found significantly higher SPSQ scores among students, as compared to employed people and pensioners; among respondents with higher education, as compared to those with lower education; among religious, as compared to non-religious people; and among practicing believers, as compared to the non-practicing. In addition, it was suggested that the SPSQ measures the SPS construct equivalently between males and females. We also found that highly sensitive respondents were more likely to report higher feelings of anxiety and deteriorated relationships during the COVID-19 pandemic.

The CFA showed that the two-factor model suggested by the EFA did not sufficiently fit with the data. Satisfactory results were found either for the complete SPSQ Scale or for its Sensory Sensitivity Subscale, suggesting that the items focusing on experiencing sensory stimuli represent a unique, though not the only aspect of the SPS. These results partly correspond to the findings of other studies focusing on validation of the Highly Sensitive Person Scale (HSPS). Though it uses a different tool, this suggestion is similar to our solution. The whole scale, as well as its Sensory Sensitivity Subscale, showed good reliability, while the correlations between the individual items of the scale were mostly low to moderate. These results suggest that the proposed instrument measures our phenomena well, even with a low number of items. Regarding the excluded items of the Sensory Sensitivity Subscale, further research is needed, as sensitivity to caffeine and a lower pain threshold are often mentioned among SPS traits. However, reactivity to stimulants might also be influenced by other internal factors. Moreover, further studies should also explore the general caffeine intake among HSP. Regarding higher sensitivity to pain, because of the highly individual nature of this item, respondents might find it too difficult to compare themselves with other people. In general, we observed the strongest factor loadings in items assessing the main senses (i.e., light, sounds, smells, taste and tactile stimuli), while reactions to other stimuli had a higher variability and thus may be more influenced by other factors. Finally, it is possible that the two items excluded from the Other Sensitivity Subscale, i.e., “lack of privacy” and “stay in nature”, might still correspond to the needs of HSP. However, in certain life situations (e.g., motherhood) these needs may not be adequately fulfilled, so HSP respondents could not properly compare themselves with the others.

Regarding sociodemographic differences, we found that women reported higher SPSQ scores than men. These findings are consistent with the original research of the authors of the HSPS [1], but they are in contrast to later theoretical presumptions, which stated that there are no gender differences in this trait [48]. Nevertheless, Aron and Aron [1] also suggest that both genders could be equally sensitive and only due to social expectations, men might be less likely to accept and admit this trait. This explanation was, however, not supported in our study in measurement invariance testing. Thus, other research approaches, e.g., using physiological measures, should be applied to get further insight into this field. In our study, SPS was also associated with education levels, as we observed higher scores among students and among respondents with higher education. Perhaps there is an association of SPS with giftedness, as suggested by some of the popular literature; however, more solid research would be needed to confirm this association. Furthermore, we observed higher SPSQ scores among religious and among practicing respondents, and this corresponds to the already proposed higher levels of spirituality in HSPs (Aron 2013). It is possible that deeper processing also leads one to a higher reflectiveness and a stronger need for meaning and transcendence in life.

We also found that SPSQ assesses the SPS construct invariantly between males and females. This decreases the probability that some undesired effects significantly contributed to the score difference between males and females. This finding also suggests that items of the SPSQ were created in an optimal way.

Finally, we found that highly sensitive respondents had a higher risk of deteriorated feelings (i.e., loneliness, threat, fear and anxiety, helplessness and decreased hope) during the first wave of the COVID-19 pandemic. The negative mental health outcomes of HSPs during the observed period may be explained by several mechanisms. First, HSPs are influenced by negative childhood experiences and poor parenting more than other people [49], which can consequently lead to higher anxiety and depression levels in adult life [5,6]. Therefore, during this time of heightened uncertainty and exposure to global stressors [50], HSPs could have been more vulnerable to negative mental health outcomes due to the experience of peritraumatic psychological distress related to the COVID-19 pandemic. Second, the pandemic has already been connected with a higher occurrence of psychosomatic problems [51]. Because HSPs are more aware of their somatic symptoms and also pay more attention to minor physiological sensations than individuals with normal sensitivity, this oversensitivity to somatic symptoms may in turn contribute back to their negative mental health outcomes.

### 4.1. Strengths and Limitations

The most important strength of this study is that it offers a new tool for measuring SPS that is short, easy to administer and has good psychometric characteristics. Second, this study offers unique insight into the associations of SPS with experiencing the COVID-19 pandemic. Third, it is based on a large sample of Czech adults that is balanced and close to national characteristics regarding gender and age. A limitation of our study is its cross-sectional design, which does not allow us to conclude on causality. The second limitation may be information bias, as our data were based on the self-reports of respondents, which can be influenced by social desirability. The third limitation is a lack of similar studies, as it does not allow us to provide a more detailed comparison. The fourth limitation is that, in the assessment of the associations of experiences related to the COVID-19 pandemic, we did not use any validated instrument. This was not then possible, because during the first wave of the pandemic, there were no validated instruments available. The last limitation is that the study is not based on representative data, so we cannot fully generalize the findings to the whole population.

### 4.2. Implications

Our findings show that highly sensitive people had more difficulties and negative feelings related to the COVID-19 pandemic. This information will be useful for psychologists, psychiatrists, psychotherapists and other helping professionals. With information about individuals’ SPS, healthcare providers and public health interventionists can further design appropriate programs to take care of the traumatized population during the COVID-19 pandemic. Furthermore, a brief psychometric tool for SPS could also be used for large-scale public health surveys in the future.

The results of the analyses showed that our new tool, the SPSQ, has good psychometric characteristics, suggesting its suitability for measuring sensory processing sensitivity. Future research should repeat these analyses on a representative adult sample and on international samples to assess possible cultural differences. It shall also explore the associations of SPS with experiences related to COVID-19 pandemic in more detail.

## 5. Conclusions

The aim of this study was to assess the psychometric properties of a newly developed tool, the Sensory Processing Sensitivity Questionnaire, and to assess selected associations with experiencing the first wave of the COVID-19 pandemic. Based on psychometric analyses, we propose a 16-item final version of the tool, with eight items covering sensory sensitivity and eight items focusing on other sensitivity aspects, i.e., life experiences and emotional sensitivity. The psychometric assessment suggested that the scale could either be used as a whole or that the Sensory Sensitivity Subscale could also be used separately. We observed higher SPSQ scores among women, students and religious respondents and higher feelings of anxiety and deteriorated relationships during the COVID-19 pandemic among highly sensitive respondents. Thus, this study offers a short tool for measuring SPS and identifies people with this trait as being potentially more vulnerable in difficult times.

## Figures and Tables

**Table 1:** Results of Exploratory Factor Analysis of one and the two-factor solutions.

		Two-Factor Solution	
	Items	FL	h2
Sensory Sensitivity Subscale			
	1. Light	0.69	0.44
	2. Sounds	0.72	0.55
	3. Smells	0.77	0.59
	4. Taste	0.88	0.71
	5. Tactile stimuli (touch, clothing, etc.)	0.75	0.59
	6. Hunger	0.52	0.36
	7. Heat	0.59	0.46
	8. Cold	0.46	0.37
Other Sensitivity Subscale			
	1. Your emotions	0.83	0.63
	2. Emotions of other people	0.74	0.52
	3. Sudden changes	0.82	0.65
	4. Your inner world	0.79	0.65
	5. The need to do many things at once	0.54	0.43
	6. Criticism	0.71	0.51
	7. The need for harmony in life	0.71	0.54
	8. The need to make decisions	0.70	0.56

Note: FL = factor loading, h2 = communalities.

**Table 2:** Goodness-of-fit indices of the SPSQ models tested in the CFA.

Fit Measure	One Factor Model	Two-Factor Model	Bi-Factor Model
*χ* ^2^	2565.761	1002.759	506.141
df	104.000	103.000	88.000
*p*-value	0.000	0.000	0.000
CFI	0.958	0.985	0.993
TLI	0.951	0.982	0.990
RMSEA	0.157 (0.152–0.162)	0.095 (0.090–0.101)	0.070 (0.065–0.076)
SRMR	0.089	0.053	0.039
*χ*^2^/*df*	24.67	9.764	5.751

**Table 3:** Descriptive statistics of the SPSQ items, correlations to composite score and between items and standard errors of items from the bi-factor model.

	M	SD	Skewness	Kurtosis	ITC	IIC	SE (SS)	SE (GS)
Sensory Sensitivity Items								
SPSQ 1 (S)	4.45	2.46	−0.32	−0.18	0.60	0.44	0.022	0.022
SPSQ 2 (S)	4.94	2.53	−0.29	−0.23	0.68	0.43	0.020	0.021
SPSQ 3 (S)	5.05	2.47	−0.21	−0.06	0.67	0.43	0.019	0.023
SPSQ 4 (S)	4.81	2.30	−0.33	0.33	0.73	0.43	0.017	0.022
SPSQ 5 (S)	4.83	2.31	−0.33	0.37	0.71	0.43	0.020	0.021
SPSQ 6 (S)	4.77	2.32	−0.18	0.12	0.58	0.44	0.023	0.021
SPSQ 7 (S)	5.32	2.39	−0.27	0.07	0.64	0.44	0.022	0.021
SPSQ 8 (S)	5.40	2.55	−0.13	−0.30	0.60	0.44	0.023	0.021
Other Sensitivity Items								
SPSQ 1 (O)	5.44	2.31	−0.13	0.03	0.69	0.43	0.030	0.023
SPSQ 2 (O)	5.11	2.16	−0.24	0.28	0.63	0.44	0.030	0.027
SPSQ 3 (O)	5.36	2.21	−0.19	0.22	0.71	0.43	0.029	0.018
SPSQ 4 (O)	5.23	2.30	−0.20	0.19	0.70	0.43	0.030	0.019
SPSQ 5 (O)	5.15	2.29	−0.23	0.13	0.64	0.43	0.031	0.018
SPSQ 6 (O)	5.45	2.30	−0.24	0.17	0.66	0.43	0.030	0.016
SPSQ 7 (O)	6.04	2.39	−0.32	0.07	0.67	0.43	0.032	0.016
SPSQ 8 (O)	5.49	2.18	−0.20	0.61	0.68	0.43	0.032	0.017

Note: ITC, corrected item–total correlation corrected for overlap of item; IIC, inter-item correlation; SE = standard error; GS = General Sensitivity factor; SS = Sensory Sensitivity factor; O = Other Sensitivity factor; M = mean; SD = standard deviation.

**Table 4:** Fit indices of the individual invariance models.

Model	*χ* ^2^	df	*p*-Value	CFI	TLI	RMEA 90% CI	SRMR
Male model	619.422	85	*p* < 0.001	0.936	0.909	0.081(0.075–0.087)	0.035
Female model	554.443	85	*p* < 0.001	0.93	0.901	0.076 (0.07–0.082)	0.037
Configural model	1173.865	170	*p* < 0.001	0.933	0.906	0.078 (0.074–0.083)	0.036
Metric model	1209.548	199	*p* < 0.001	0.933	0.919	0.073 (0.069–0.077)	0.038
Scalar model	1287.684	212	*p* < 0.001	0.928	0.919	0.073 (0.069–0.077)	0.04
Strict model	1438.483	228	*p* < 0.001	0.919	0.915	0.074 (0.071–0.078)	0.043

Note: *χ*^2^ = chi-squared, df = degrees of freedom, CFI = comparative fit index, TLI = Tucker–Lewis Index, RMSEA = Root Mean Square of Approximation, SRMR = Standardized Root Mean Squared Residual. In RMSEA, values in brackets represent 90% confidence intervals.

**Table 5:** Sociodemographic characteristics of the first sample with means and standard deviations of the SPSQ total and Sensory Sensitivity Scale scores used to test group differences.

	Total	Total Score	Group Differences	Sensory Sensitivity Subscale	Group Differences
	N (%)	M (SD)		M (SD)	
**Gender:**			W = 5800, *p* < 0.001, *r* = 0.27		W = 54,906, *p* < 0.001, *r* = 0.17
1. Female	967 (50.4%)	5.5 (1.6)		5.2 (1.8)	
2. Male	952 (49.6%)	4.9 (1.6)		4.7 (1.8)	
**Family status:**			n.s		n.s.
1. No relationship	817 (42.6%)	5.2 (1.6)		4.9 (1.9)	
2. In a relationship	1102 (57.4%)	5.2 (1.6)		5.0 (1.8)	
**Religious attendance:**			W = 44,270, *p* = 0.014, *r* = 0.07		W = 44,101, *p* = 0.017, *r* = 0.07
1. Once a week or more	64 (5.18%)	5.7 (1.4)		5.5 (1.7)	
2. Less than once a week	1171 (94.8%)	5.2 (1.6)		4.9 (1.8)	
**Economic status:**			*Z* = −3.24, *p* = 0.018, $\hat{A}$ = 0.4		1 vs. 6: (*Z* = −3.47, *p = 0.008*, $\hat{A}$ = 0.39); 4 vs. 6: (*Z* = −3.08, *p* = 0.031, $\hat{A}$ = 0.41)
1. Employed	915 (47.8%)	5.1 (1.6)		4.8 (1.8)	
2. Entrepreneur	108 (5.64%)	5.4 (1.4)		5.2 (1.6)	
3. In household/without work	88 (4.59%)	5.4 (1.7)		5.1 (2.0)	
4. Pensioner	622 (32.5%)	5.2 (1.6)		5.0 (1.8)	
5. Maternity leave	83 (4.33%)	5.4 (1.8)		5.0 (1.9)	
6. Student	100 (5.22%)	5.6 (1.4)		5.5 (1.6)	
**Education:**			2 vs. 5: t(498) = 3.71, *p* = 0.002, $\hat{A}$ = 0.45		n.s.
1. Elementary school	163 (8.50%)	5.2 (1.8)		4.9 (1.8)	
2. Vocational school or non-maturity high school	778 (40.6%)	5.0 (1.7)		4.8 (2.0)	
3. High school	672 (35.0%)	5.3 (1.4)		5.0 (1.7)	
4. Higher vocational school or University bachelor	61 (3.18%)	5.6 (1.2)		5.3 (1.5)	
5. College	244 (12.7%)	5.4 (1.4)		5.1 (1.5)	
**Faith:**			W = 144,738, *p* < 0.001, *r* = 0.1		W = 138,508, *p* < 0.001, *r* = 0.12
1. Religious	355 (34.2%)	5.7 (1.6)		5.5 (1.7)	
2. Non-religious	684 (65.8%)	5.2 (1.6)		5.0 (1.7)	
M(SD)		5.18 (1.6)		4.95 (1.8)	
**SPSQ** Median		5.19		5	
Min, Max		0–10		0–10	

Note: M = mean, SD = standard deviation, N = number of subjects, n.s. = non-significant, group comparisons were analyzed by using non-paramedic analysis of variance, Min = minimum value, Max = maximum value, t-value refers to the result of the Games–Howell test, W statistic reflects the result of Mann–Whitney U test, *Z* value indicates the result of the Dunn test post hoc test, $\hat{A}$ = Vargha and Delaney effect size, *r* = rank/bi-serial correlation, indicating Mann–Whitney U test effect size.

**Table 6:** Associations of the SPSQ total score and its sensory component with deteriorated feelings, deteriorated relationships and deteriorated structure of the day during the COVID-19 pandemic.

		Deteriorated Feelings				
		Loneliness	Threat	Fear and Anxiety	Helplessness	Hope
SPSQ	**Crude**	1.47 (1.23–1.76) ***	1.61 (1.38–1.88) ***	1.84 (1.56–2.19) ***	1.52 (1.30–1.80) ***	1.50 (1.20–1.88) ***
SPSQ Sensory Subscale		1.23 (1.03–1.48) *	1.43 (1.24–1.67) ***	1.55 (1.32–1.83) ***	1.33 (1.13–1.57) ***	1.24 (1.00–1.56)
SPSQ	**Adjusted**	1.33 (1.11–1.61) ***	1.56 (1.34–1.83) ***	1.68 (1.42–2.01) ***	1.41 (1.19–1.67) ***	1.40 (1.11–1.76) ***
SPSQ Sensory Subscale		1.14 (0.95–1.37)	1.40 (1.20–1.64) ***	1.44 (1.22–1.71) ***	1.25 (1.07–1.48) **	1.17 (0.94–1.48)
		**Deteriorated Relationships**			**Deteriorated Structure of a Day**	
		**Partner**	**Children**	**Other Persons in the Household**		
SPSQ	**Crude**	1.68 (1.26–2.26) ***	1.56 (1.11–2.22) **	1.60 (1.13–2.29) **	1.24 (1.08–1.43) ***	
SPSQ Sensory Subscale		1.47 (1.09–2.00) **	1.44 (1.02–2.07) *	1.25 (0.89–1.80)	1.14 (0.99–1.31)	
SPSQ	**Adjusted**	1.58 (1.17–2.16) ***	1.51 (1.08–2.16) *	1.48 (1.03–2.16) *	1.22 (1.06–1.41) **	
SPSQ Sensory Subscale		1.36 (1.00–1.88) *	1.40 (0.99–2.03)	1.12 (0.79–1.63)	1.12 (0.97–1.29)	

Note: The independent variable was standardized to Z-score. Adjusted models included age, gender and socioeconomic status as covariates. Results are reported in odds ratios (ORs) with 95% confidence intervals (95% CIs); *** *p* < 0.001, ** *p* < 0.01, * *p* < 0.05; bold values represent significant results after Bonferroni correction.

## Data Availability

Data, code and other materials supporting the results of this study can be found on the Open Science Framework webpage (https://doi.org/10.17605/OSF.IO/SER9H), (accessed on 6 December 2021) reference number SER9H.

## Supplementary Materials

The following are available online at https://www.mdpi.com/article/10.3390/ijerph182412962/s1, File S1: Sensory Processing Sensitivity Questionnaire (SPSQ).
