# Supplementary material for: Sensory Processing Sensitivity Questionnaire: A Psychometric Evaluation and Associations with Experiencing the COVID-19 Pandemic
Source: Int J Environ Res Public Health. 2021 Dec 8;18(24):12962. doi: 10.3390/ijerph182412962 (PMC8700833; doi:10.3390/ijerph182412962)
Supplement: Supplementary file 1 [file ijerph-18-12962-s001.zip › ijerph-1415598-supplementary.pdf]

Sensory Processing Sensitivity Questionnaire (SPSQ)

Please indicate to what extent you think that compared to other people you are sensitive to the following stimuli, conditions or experiences

0 = compared to others, I am not sensitive to them at all
5 = about the same as the people around me
10 = much more sensitive than the people around me

		0	1	2	3	4	5	6	7	8	9	10
1	Light											
2	Sounds											
3	Smells											
4	Taste											
5	Tactile stimuli – touch, clothing, etc.											
6	Hunger											
7	Heat											
8	Cold											
9	Your emotions											
10	Emotions of other people											
11	Sudden changes											
12	Your inner world											
13	The need to do many things at once											
14	Criticism											
15	The need for harmony in life											
16	The need to make decisions											

Sensory Sensitivity subscale: items 1-8
Other Sensitivity subscale: items 9-16
